# Diagnostic Accuracy of Contrast-Enhanced Ultrasound (CEUS) in the Detection of Muscle-Invasive Bladder Cancer: A Systematic Review and Diagnostic Meta-Analysis

**DOI:** 10.3390/curroncol31020060

**Published:** 2024-02-01

**Authors:** Antonio Tufano, Davide Rosati, Martina Moriconi, Valerio Santarelli, Vittorio Canale, Stefano Salciccia, Alessandro Sciarra, Giorgio Franco, Vito Cantisani, Giovanni Battista Di Pierro

**Affiliations:** 1 Department of Maternal-Infant and Urological Sciences, “Sapienza” Rome University, Policlinico Umberto I Hospital, 00185 Rome, Italy; 2 Department of Radiology, Oncology and Pathology, University La Sapienza of Rome, 00185 Rome, Italy

**Keywords:** contrast-enhanced ultrasound (CEUS), bladder cancer, accuracy, muscle invasive, ultrasound, meta-analysis

## Abstract

Background: Contrast-enhanced ultrasound (CEUS) is a diagnostic tool that is gaining popularity for its ability to improve overall diagnostic accuracy in bladder cancer (BC) staging. Our aim is to determine the cumulative diagnostic performance of CEUS in predicting preoperative muscle invasiveness using a comprehensive systematic review and pooled meta-analysis. Methods: A systematic review until October 2023 was performed according to the Preferred Reporting Items for Systematic Reviews and Meta-Analyses (PRISMA) statement. Patients with BC suspicion were offered CEUS before the transurethral resection of the bladder tumor (TURBT). The diagnostic performance of CEUS was evaluated based on non-muscle-invasive bladder cancer (NMIBC) vs. muscle-invasive bladder cancer (MIBC) confirmed at the final histopathological examination after TURBT. The outcomes were determined through pooled sensitivity, specificity, pooled positive likelihood ratio (PLR+), negative likelihood ratio (PLR−), and area under the summary receiver operating characteristic (SROC) along with their respective 95% confidence intervals (CI). Results: Overall, five studies were included. In these studies, a total of 362 patients underwent CEUS prior to TURBT. The pooled sensitivity and specificity were 0.88 (95% CI: 0.81–0.93) and 0.88 (95% CI: 0.82–0.92), respectively. SROC curve depicted a diagnostic accuracy of 0.94 (95% CI: 0.81–0.98). The pooled PLR+ and PLR− were 7.3 (95% CI: 4.8–11.2) and 0.14 (95% CI: 0.08–0.23), respectively. Conclusions: Our meta-analysis indicates that CEUS is highly accurate in the diagnosis and staging for BC. Beyond its accuracy, CEUS offers the advantage of being a cost-effective, safe, and versatile imaging tool.

## 1. Introduction

Bladder cancer (BC) represents the tenth most common cancer worldwide [1]. Men account for the majority of new cases, although women exhibit higher disease-specific mortality [2]. BC is classified according to the TNM staging, which considers the extent of invasion into the bladder wall (T), the presence of lymph node involvement (N), and whether it is spread to nearby or distant organs (M) [3]. The precise evaluation of muscular invasion is crucial for determining the appropriate treatment, as patients with non-muscle-invasive bladder cancer (NMIBC) and muscle-invasive bladder cancer (MIBC) necessitate different therapeutic approaches. In this scenario, computed tomography (CT) and multiparametric magnetic resonance imaging (mpMRI) are non-invasive diagnostic techniques, each exhibiting variable degrees of accuracy in staging BC [4,5]. More specifically, a standardized methodology for imaging and reporting mpMRI in BC patients was developed by Panebianco et al. in 2018 with the Vesical Imaging-Reporting and Data System (VI-RADS) score [6]. Despite being relatively new, two diagnostic meta-analyses evaluating VI-RADS have been conducted, pooling accuracy data [7,8]. These analyses demonstrate a highly promising and excellent performance in distinguishing between NMIBC and MIBC.

In the diagnostic work of BC, ultrasound examination (US) represents a well-accepted, non-invasive, and cost-effective diagnostic tool. The B-mode and Doppler US can reveal the tumor and supply blood in the base of the tumor, yet it has a low sensibility in providing a clear depiction of the muscularis layer. However, the advent of contrast-enhanced ultrasound (CEUS) and the new generation of ultrasound contrast agents (UCAs) have resulted in overcoming the inherent limitations associated with conventional B-mode and Doppler US, offering unprecedented insights into parenchymal microvasculature [9,10,11]. Moreover, real-time evaluation stands as one of the primary advantages of employing CEUS. Conversely, with CT or mpMRI, it is imperative to determine the ideal acquisition time in order to achieve an optimal differentiation between the lesion and the bladder wall. To guide and standardize the application of CEUS in the urinary bladder, collaborative efforts have been initiated. In particular, the EFSUMB–WFUMB collaboration in 2018 has produced comprehensive guidelines and clinical practice recommendations for CEUS [12]. However, despite the promising implications of incorporating CEUS into the therapeutic uro-oncologic algorithm of BC, several issues remain unresolved: first, ensuring reproducibility across different CEUS readers with varying levels of experience; second, establishing appropriate threshold cut-off score criteria for defining muscle invasiveness. Addressing these unmet needs is crucial for providing definitive guidance before embarking on further dedicated clinical trials and investigations to assess the predictive value of CEUS in determining muscle invasion in BC patients. To take the initial step toward this goal, we conducted an updated and comprehensive systematic review and metanalysis of the literature, incorporating all available international experiences that validate CEUS in the pre-transurethral resection of bladder tumor (TURBT) setting for muscle-invasive bladder cancer (MIBC) determination.

## 2. Materials and Methods

### 2.1. Search Strategy

We conducted a comprehensive search of the PubMed, Embase, and Web of Science databases without language restrictions, covering the period from inception to October 2023, in accordance with the Preferred Reporting Items for Systematic Reviews and Meta-Analyses (PRISMA) guidelines [13]. The search strategy included the following Medical Subject Headings (MeSH AND PubMed controlled vocabulary) and free text keywords: “CEUS” [All Fields] AND “urinary bladder neoplasms” [MeSH Terms], “CEUS” [All Fields] AND (“bladder s” [All Fields] OR “urinary bladder” [MeSH Terms] OR (“urinary” [All Fields] AND “bladder” [All Fields]) OR “urinary bladder” [All Fields] OR “bladder” [All Fields] OR “bladders” [All Fields]), “bladder’s” [All Fields] OR “urinary bladder” [MeSH Terms] OR (“urinary” [All Fields] AND “bladder” [All Fields]) OR “urinary bladder” [All Fields] OR “bladder” [All Fields] OR “bladders” [All Fields]. Additionally, we reviewed the reference lists of included papers to identify any further pertinent studies.

### 2.2. Inclusion and Exclusion Criteria

The study’s eligibility criteria were determined based on the Population, Intervention, Comparison, Outcome, and Study Design (PICOS) framework.

– Population: patients with suspicion of BC.
– Intervention: CEUS of the urinary bladder before TURBT.
– Comparator: final histopathological examination.
– Outcome: evaluation of CEUS diagnostic accuracy, including sensitivity, specificity, accuracy, and likelihood ratio.
– Study Design: prospective and retrospective cohort studies.

Exclusion criteria included CEUS and pathological findings indicating a cancer other than bladder urothelial carcinoma, or as benign neoformations. Abstracts, case reports, and studies with overlapping patient data were excluded from consideration. The exploration of eligibility criteria was conducted by two authors (A.T. and V.C.). Data review and extraction were independently performed by two investigators (A.T. and D.R), with any potential conflicts resolved through discussion or consultation with a third investigator (G.B.D.P.).

### 2.3. Methodological Quality Assessment

Each study’s quality was assessed using the Quality Assessment of Diagnostic Accuracy Studies–2 (QUADAS–2) tool [14]. The QUADAS–2 framework comprises four domains: (1) patient selection, (2) index test, (3) reference standard, and (4) flow and timing. Within each domain, the risk of bias and concerns regarding applicability were examined and categorized as low, high, or unclear risk. The outcomes of the quality assessment were employed for descriptive purposes, offering an evaluation of the overall quality of the included studies and exploring potential sources of heterogeneity.

### 2.4. Data Collection

The evaluation of diagnostic performance was based on NMIBC vs. MIBC; for each endpoint, true positive (TP), false positive (FP), true negative (TN), and false negative (FN) values were extracted, whenever available, from each study and recorded in 2 × 2 contingency tables. Subsequently, sensitivity, specificity, positive predictive value (PPV), and negative predictive value (NPV) for each study were calculated. Additionally, the area under the summary receiver operating characteristic (SROC) curve was computed. Descriptive variables, such as study design, year, number of patients, age, tumor size, dose and type of contrast injected, and number of readers, were also extracted.

### 2.5. Statistical Analysis

The outcomes were determined through pooled sensitivity, specificity, pooled positive likelihood ratio (PLR+), negative likelihood ratio (PLR−), and area under the summary receiver operating characteristic (SROC) along with their respective 95% confidence intervals (CI).

In our analyses, we adopted the bivariate random-effects model since this method is the preferred approach in investigating the diagnostic accuracy, particularly in the presence of heterogeneity [15]. Heterogeneity was evaluated through the Q test and I^2^ statistic. I^2^ values were categorized as follows: I^2^ ≤ 25% denoting low heterogeneity, 25% < I^2^ ≤ 50% indicating mild heterogeneity, 50% < I^2^ ≤ 75% reflecting moderate heterogeneity, and I^2^ > 75% suggesting high heterogeneity. All the statistical analyses were conducted using STATA software (version 18.0; Stata Corporation, College Station, TX, USA). Statistical analysis was two-sided and statistical significance was set at *p* < 0.05.

## 3. Results

### 3.1. Literature Search

**Figure 1:**
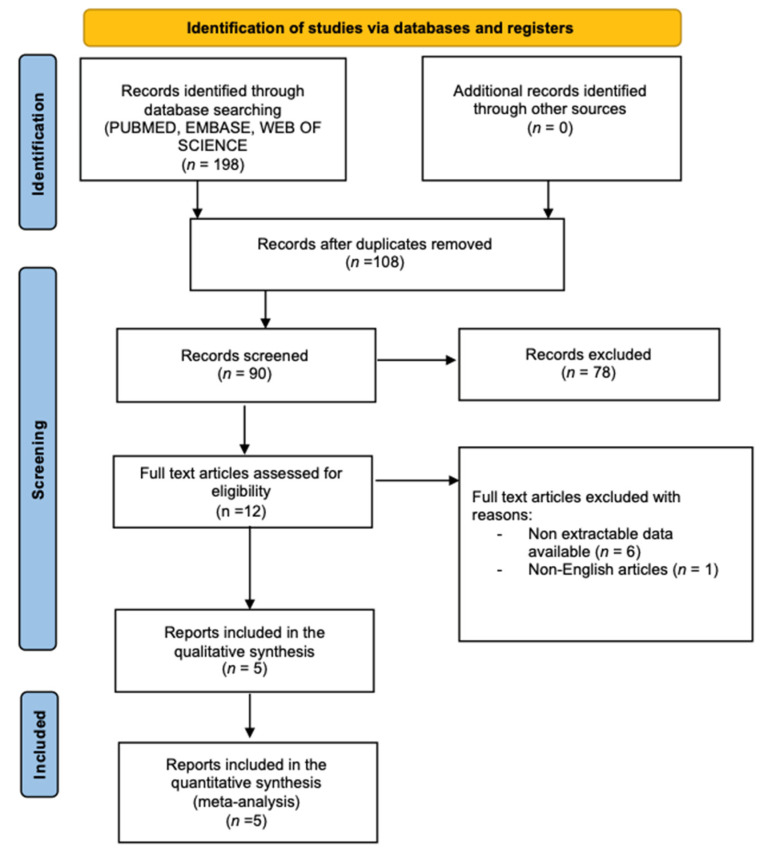
Study flow chart.

The study selection process is illustrated in Figure 1 using the PRISMA flow chart. The initial search yielded 198 studies, with 90 excluded due to duplication. Following the application of selection criteria, an additional 103 records were excluded. Finally, *n* = 5 studies were included in the systematic review and metanalysis [16,17,18,19,20]. No further articles were identified in the reference section.

### 3.2. Study Characteristics

The articles included in the analysis were conducted in China (*n* = 3), Italy (*n* = 1), and India (*n* = 1), covering a total of 423 suspected BC patients with a mean age ranging from 61 to 69 years. Among these, 362 patients underwent CEUS. At the histopathological examination, 178 cases were identified as MIBC, and 184 were NMIBC. Table 1 provides a summary of the principal characteristics of the studies included. The mean tumor size was reported in 3/5 studies, ranging from 2.7 to 3.0 cm. Each study also documented the doses of contrast agent (SonoVue, Bracco) administered, with quantities ranging from 1.0 mL to 2.4 mL. Moreover, *n* = 3 studies relied on two radiologist readers, where one had a single reader, and one study did not provide information on the exact number of readers. 

### 3.3. Methodology Quality Assessment 

As per the methodological evaluation outlined in the QUADAS-2 checklist, the studies uniformly adopted a prospective design, affirming the overall quality of the included studies. Consecutive patient enrollment was a consistent practice across all studies, with cystoscopy and/or transurethral resection of bladder tumor (TURBT) serving as the reference standard. The risks associated with case selection, trial assessments, gold criteria, and clinical applicability were all deemed low. Further elaboration of these findings can be found in Supplementary Table S1.

### 3.4. Data Synthesis and Analysis 

**Figure 2:**
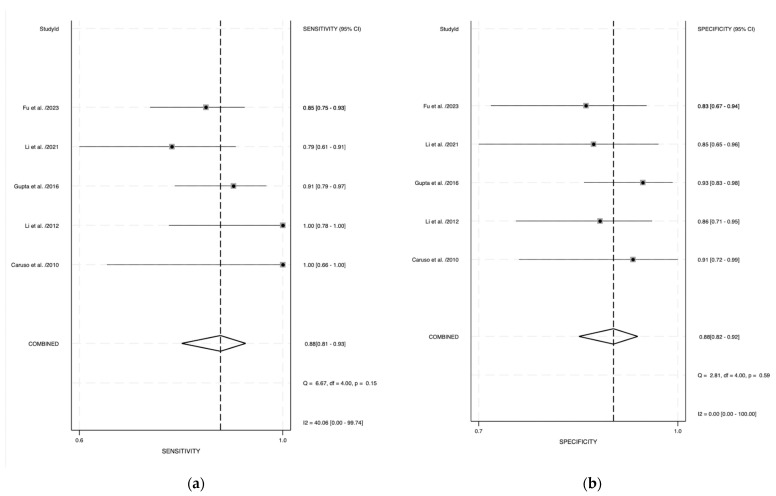
(**a**) Forest plot of pooled sensitivity of CEUS in detecting muscle invasion involvement. (**b**) Forest plot of pooled specificity of CEUS in detecting muscle invasion involvement [16,17,18,19,20].

**Figure 3:**
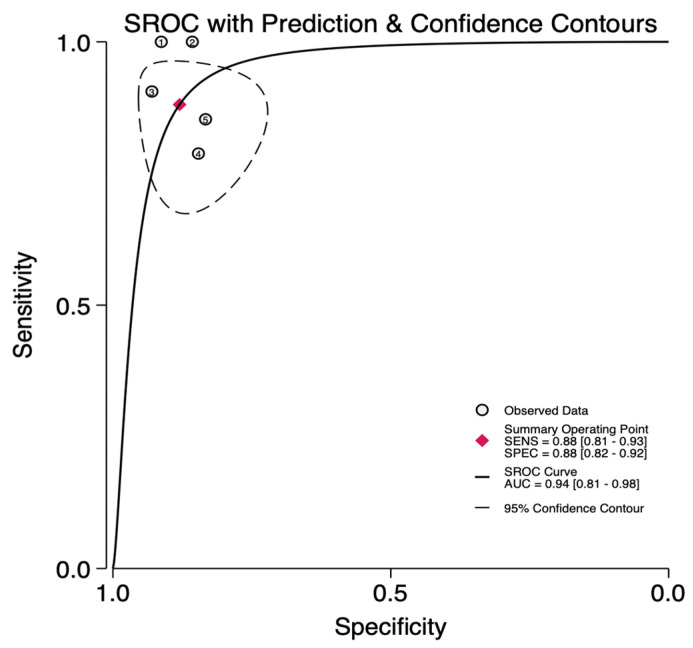
SROC for diagnostic performance of studies using CEUS predicting MIBC.

Among the included studies, pooled sensitivity and specificity were 0.88 (95% CI: 0.81–0.93) and 0.88 (95% CI: 0.82–0.92), respectively (Figure 2a,b). SROC curve depicted a diagnostic accuracy of 0.94 (95% CI: 0.81–0.98) (Figure 3), while the pooled PLR+ and PLR− were 7.3 (95% CI: 4.8–11.2) and 0.14 (95% CI: 0.08–0.23), respectively. The I^2^ for the pooled sensitivity and specificity were 40% and 0%, respectively, indicating a low-mild heterogeneity between the studies.

**Figure 4:**
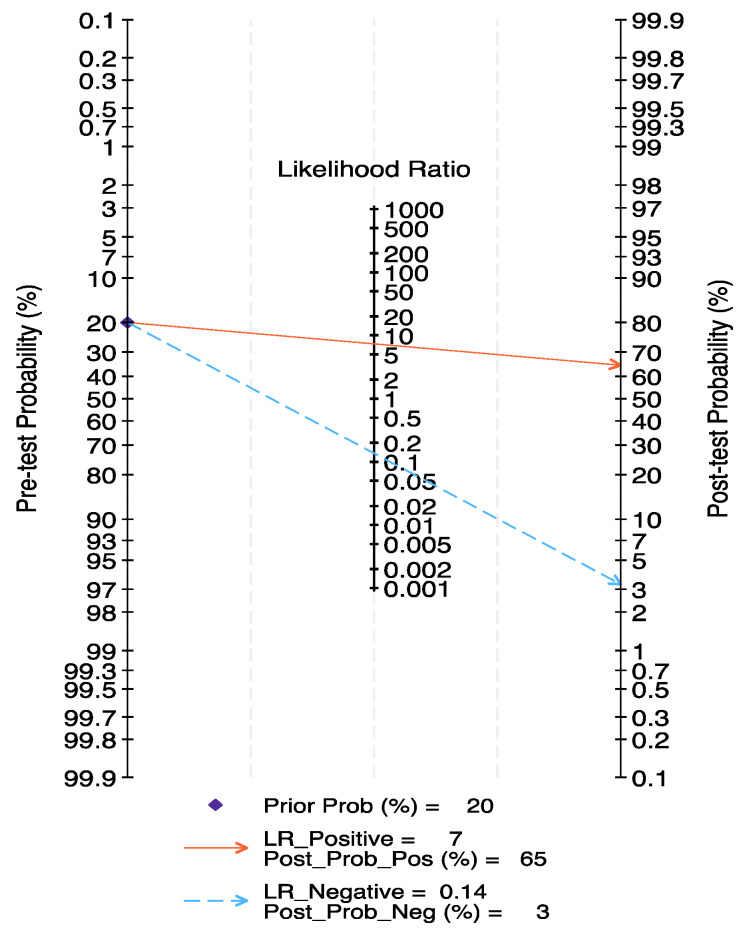
Fagan nomogram reflecting pre- and post-test probability estimation for clinical utility of CEUS in predicting MIBC.

Fagan diagram was drawn according to the Bayes principle; given a pre-test probability of 20%, the PLR+ increased from 20% to 65%, while the PLR− decreased to 3% (Figure 4). 

### 3.5. Publication Bias

**Figure 5:**
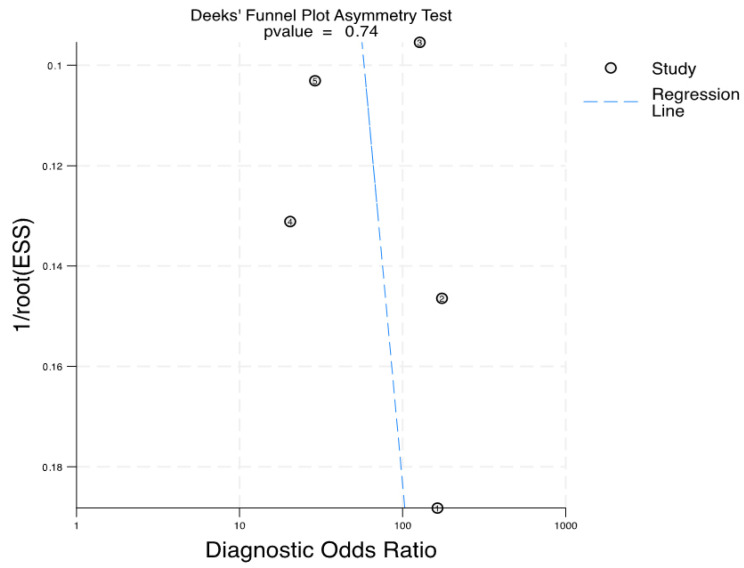
Deeks’ funnel plot asymmetry test for the assessment of publication bias.

The Deeks’ funnel plot was employed to identify any systematic error related to publication bias. With a *p*-value of 0.74, the distribution of included studies on both sides of the regression line suggests no significant evidence of publication bias, *p* = 0.74 (Figure 5).

## 4. Discussion

CEUS is a versatile and well-tolerated diagnostic tool, rapidly expanding its applications in multiple organs [21,22,23]. Several versions of guidelines discussing the hepatic applications of CEUS have been published since 2004 by the European Federation of Societies for Ultrasound in Medicine and Biology (EFSUMB) and the World Federation for Ultrasound in Medicine and Biology (WFUMB) [24]. However, the concrete application in bladder pathologies is still under investigation. The urinary bladder is characterized by its complexity and unique challenges in imaging and prompts a focused inquiry into the adaptability and efficacy of CEUS in this specific context. A first step toward addressing this issue was introduced by EFSUMB within the context of “non-hepatic uses of contrast-enhanced ultrasound” [25].

BCs are commonly categorized as either NMIBC or MIBC. NMIBCs, constituting around two-thirds of BCs, are tumors that affect only the immediate epithelial layer of cells (CIS and Ta) or penetrate the subepithelial connective tissue (T1). In contrast, MIBCs, accounting for one-third of cases, include tumors that infiltrate the muscularis propria (T2), penetrate through the muscularis propria to involve the perivesical tissue (T3), or extend into adjacent pelvic or abdominal organs (T4). As a result, tumors classified as Ta-T1 are considered NMIBCs, while those categorized as T2–T4 are considered MIBCs. Defining these criteria is crucial because prognosis and the choice of appropriate management strategies heavily rely on accurately staging the tumor. Therefore, we aimed to provide pooled data on the value of CEUS in distinguishing NMIBC vs. MIBC, and our analyses resulted in several noteworthy observations.

The first reassuring finding in our analysis was that CEUS revealed an accuracy of 0.94 (95% CI: 0.81–0.98), with a sensitivity of 0.88 (95% CI: 0.81–0.93) and a specificity of 0.88 (95% CI: 0.82–0.92) in discriminating NMIBC vs. MIBC. Notably, Li and colleagues found that CEUS exhibited sensibility and specificity comparable to mpMRI in BC staging and grading, underscoring its value as a preoperative imaging modality for delineating tumor invasion [19]. Moreover, the accuracy achieved through the combined diagnosis of CEUS and MRI + diffusion-weighted imaging (DWI) was higher than that with the single diagnostic method [19]. Hence, the combination of CEUS and MRI + DWI proved to be a viable and effective approach for the clinical diagnosis of BC. Interestingly, the LRs, which represent comprehensive indicators reflecting the accuracy values of diagnostic tests, were notably robust in our study, with a positive LR (PLR+) of 7.3 (95% CI: 4.8–11.2) and a negative LR (PLR−) 0.14 (95% CI: 0.08–0.23), providing compelling diagnostic evidence. These results collectively highlight the robust diagnostic performance of CEUS in the assessment of BC in both NMIBC and MIBC patients. This distinction should guide future research recommendations where the utilization of CEUS may direct patients towards invasive and radical interventions, such as neoadjuvant chemotherapy + radical cystectomy. Furthermore, CEUS can serve as a noninvasive approach for postoperative follow-up or in high-risk NMIBC patients, suggesting the potential to avoid early repeated resection (Re-TURB) offering valuable imaging information and with consequential socioeconomic implications and impact on health-care–related costs. Second, we would like to emphasize the potential role of CEUS as a first-line approach in patients with hematuria, as conventional US techniques, such as greyscale US and color Doppler ultrasound (CDUS), exhibited limited effectiveness in diagnosing malignant disease [26]. Nonetheless, previous studies concluded that tumor vascularity and lesion size detected by CDUS did not align with tumor stage and histological grade [27]. As previously discussed, CEUS emerges as a valuable tool to overcome these limitations, enabling not only detection but also tumor staging and grade in BC. However, in the evaluation of tumor grade, some considerations should be made. Notably, previous studies assessing the potential role of CEUS in predicting BC tumor grade (high grade vs. low grade) have been proposed [18,28]. In a study by Gupta et al., CEUS was employed to predict the T stage and BC grade. Here, the sensitivity of CEUS in diagnosing BC staging was higher than its sensitivity in grading BC [18]. These analyses were based on time–intensity curves (TICs) extracted from the region of interest positioned within the lesion and in the closest bladder wall. However, we feel that caution should be reserved in interpreting these results since the software employed for quantitative analysis of CEUS differs among various manufacturers. Several proprietary software options are available, and the recorded TIC values can vary based on the specific machine, probe, signal gain settings, and software utilized [29]. Additionally, the authors observed that the quantity and dilution of the contrast agent may also have an impact on TIC values [30]. Although these studies suggest promising outcomes, due to the limited availability of data, it may be inappropriate to apply these enhancement patterns to the global population, as the incidence of urothelial carcinoma varies worldwide due to racial, sex, biological, and clinical differences [31,32,33]. 

Third, the significance of CEUS increases in patients where endoscopy examinations pose a challenge (i.e., patients with severe urethra-stenosis) or in patients with absolute contraindications for mpMRI. Moreover, CEUS has demonstrated its ability to enhance the differentiation between vascularized tumors, characterized by distinct enhancement patterns, and non-enhancing clots [34]. Hence, this feature is particularly relevant in cases of hematuria, where conventional B-mode and Doppler ultrasound findings may lack clarity. Nonetheless, three-dimensional contrast-enhanced ultrasound (3D CEUS) imaging is an innovative medical imaging technique that spatially displays images from various visual angles and uses reflections of microbubbles to clearly depict blood vessels [17]. Despite its recent introduction, 3D CEUS imaging has emerged as a valuable tool for bladder pathologies [34]. These considerations are especially relevant now, considering the increasing body of evidence that not only investigates the diagnostic performance of CEUS but also explores potential novel implications in the management of BC. Taken together, our results strongly indicate that CEUS can become a useful, non-invasive examination in the future for the differential diagnosis of NMIBC vs. MIBC. These considerations now appear to be particularly timely as a complementary growing body of evidence is emerging, exploring not solely the diagnostic performance indexes of CEUS itself but also the potential novel implications, which could derive from the internalization of a reliable preoperative staging tool in the decision-making process of daily urological practice. 

To the best of our knowledge, this is the first systematic review and meta-analysis that assesses the diagnostic performance of CEUS focusing on the evaluation of the muscularis–mucosa invasion. Nonetheless, our study is not without limitations. Firstly and more importantly, the intrinsic limitations of CEUS, such as the patient’s constitution or intestinal interposition should be considered. Secondly, the inclusion of a small number of studies (*n* = 5), with limited sample sizes, represents a further limitation of this review. Thirdly, possible confounders such as tumor sizes and the number of readers may reflect different accuracy rates. Fourth, a limit of CEUS results in low accuracy in determining the lamina propria invasion. This thin layer tends to disappear with bladder overdistension, making it particularly challenging to visualize in female patients and those with thin bladders. Hence, optimum bladder filling is of primary importance. To address this limitation, we consolidated Ta and T1 lesions into the NMIBC category. Despite these voids, our study represents the most robust evidence available, given the current lack of high-quality, well-designed, and adequately powered longitudinal studies in this field.

## 5. Conclusions

Our meta-analysis indicates that CEUS is highly accurate in the diagnosis and staging for BC, demonstrating specificity and sensitivity levels in distinguishing between Ta-T1 vs. ≥T2 stage comparable to reference standard methods. Beyond its accuracy, CEUS offers the advantage of being a more cost-effective, safe, and versatile imaging modality compared to MRI or CT. Future larger prospective studies are warranted. 

## Figures and Tables

**Table 1:** Study characteristics.

Author	Year	Country	Study Design	Patients, *N*	Male (%)	Age, Mean (SD)	Tumor Size, cm Mean (SD)	Reference	Ultrasound Contrast Agent, mL	Readers, *N*	Inter-Reader Agreement
Caruso et al. [16]	2010	Italy	Prospective	34	94.1	61 ± 8.4	3.0 ± 1.1	TURBT	SonoVue, 2.4 mL	2	0.917
Li et al. [17]	2012	China	Prospective	60	75	62 ± 13	2.9 ± 1.2	TURBT	SonoVue, 1.2 mL	2	0.914
Gupta et al. [18]	2016	India	Prospective	110	87.3	60 (median)	N.R.	TURBT	SonoVue, 2.4 mL	1	-
Li et al. [19]	2021	China	Prospective	59	NR	69 + 12	N.R.	TURBT	Sonovue 1.2 mL	N.R.	N.R.
Fu et al. [20]	2023	China	Prospective	160	66.2	52 ± 4.9	2.7 ± 1.6	TURBT	SonoVue, 1.0 mL	2	N.R.

SD = Standard deviation; TURBT = transurethral resection of bladder tumor; N.R. = not reported.

## Supplementary Materials

The following supporting information can be downloaded at: https://www.mdpi.com/article/10.3390/curroncol31020060/s1, Table S1: Quality assessment of the studies according to QUADAS-2.
