# Supplementary material for: Diagnostic Accuracy of Contrast-Enhanced Ultrasound (CEUS) in the Detection of Muscle-Invasive Bladder Cancer: A Systematic Review and Diagnostic Meta-Analysis
Source: Curr Oncol. 2024 Feb 1;31(2):818–27. doi: 10.3390/curroncol31020060 (PMC10888477; doi:10.3390/curroncol31020060)
Supplement: Supplementary file 1 [file curroncol-31-00060-s001.zip › curroncol-2831320-supplementary.pdf]

Supplementary Table 1. Quality assessment of the studies according to QUADAS-2.

Study	RISK OF BIAS				APPLICABILITY CONCERNS		
	PATIENT SELECTION	INDEX TEST	REFERENCE STANDARD	FLOW AND TIMING	PATIENT SELECTION	INDEX TEST	REFERENCE STANDARD
Caruso 2010 [16]	☺	☺	☺	☺	☺	☺	☺
Li 2012 [17]	☺	☺	☺	☺	☺	☺	☺
Gupta 2016 [18]	☺	☺	☺	☺	☺	☺	☺
Li 2021 [19]	☺	☺	☺	☺	☺	☺	☺
Fu 2023 [20]	☺	☺	☺	☺	☺	☺	☺

☺ Low Risk
